# Understanding the Evolving Role of Voluntary Medical Male Circumcision as a Public Health Strategy in Eastern and Southern Africa: Opportunities and Challenges

**DOI:** 10.1007/s11904-022-00639-5

**Published:** 2022-12-02

**Authors:** Anna Bershteyn, Edinah Mudimu, Ingrida Platais, Samuel Mwalili, James E. Zulu, Wiza N. Mwanza, Katharine Kripke

**Affiliations:** 1 Department of Population Health, New York University Grossman School of Medicine, 227 East 30th Street, New York, NY 10016 USA; 2 Department of Decision Sciences, College of Economic and Management Sciences, University of South Africa, Pretoria, Gauteng South Africa; 3 Strathmore Institute of Mathematical Sciences, Strathmore University, Nairobi, Kenya; 4 Zambia Field Epidemiology Training Program, Workforce Development Cluster, Zambia National Public Health Institute, Lusaka, Zambia; 5 Directorate of Public Health and Research, Ministry of Health, Lusaka, Zambia; 6 Avenir Health, Takoma Park, MD USA

**Keywords:** Male circumcision, HIV prevention, Sexually transmitted infections, Mathematical modeling, Cost-effectiveness, Sub-Saharan Africa

## Abstract

**Purpose of Review:**

Voluntary male medical circumcision (VMMC) has been a cornerstone of HIV prevention in Eastern and Southern Africa (ESA) and is credited in part for declines in HIV incidence seen in recent years. However, these HIV incidence declines change VMMC cost-effectiveness and how it varies across populations.

**Recent Findings:**

Mathematical models project continued cost-effectiveness of VMMC in much of ESA despite HIV incidence declines. A key data gap is how demand generation cost differs across age groups and over time as VMMC coverage increases. Additionally, VMMC models usually neglect non-HIV effects of VMMC, such as prevention of other sexually transmitted infections and medical adverse events. While small compared to HIV effects in the short term, these could become important as HIV incidence declines.

**Summary:**

Evidence to date supports prioritizing VMMC in ESA despite falling HIV incidence. Updated modeling methodologies will become necessary if HIV incidence reaches low levels.

## Introduction

Male circumcision is one of the oldest and most commonly practiced surgical procedures worldwide. Voluntary male medical circumcision (VMMC), circumcision of boys and men by a trained medical provider with the recipient’s consent and without undue influence, has been shown to significantly reduce HIV acquisition among heterosexual men [1–3]. The past two decades have seen major investments in VMMC in Eastern and Southern Africa (ESA), home to one-third of all people living with HIV (PLHIV). VMMC is now a cornerstone of HIV prevention in 15 ESA countries with the highest HIV prevalence and low male circumcision prevalence prior to the mid-2000s: Botswana, Eswatini, Ethiopia, Kenya, Lesotho, Malawi, Mozambique, Namibia, Rwanda, South Africa, South Sudan, Uganda, Tanzania, Zambia, and Zimbabwe.

These ESA priority countries are now showing steady declines in the rates of new HIV infections, driven by a combination of VMMC and antiretroviral therapy (ART) scale-up. As a “one-and-done” procedure that gives lifelong HIV prevention, VMMC has been found in numerous economic evaluations to be one of the most cost-effective HIV interventions available in ESA [4–9]. However, as with any prevention measure, the cost-effectiveness of VMMC declines as HIV incidence in the population falls.

In this review, we summarize the evidence on VMMC’s risks and benefits, methods used to translate these benefits into impact and cost-effectiveness estimates, and the current scope and shortcomings of these methods. We review the current state of evidence as to whether VMMC will remain cost-effective and high priority for HIV budget allocation in the context of declining HIV incidence. Lastly, we discuss how declining HIV incidence may shift the scope of VMMC decision analyses in ESA, including more detailed consideration of non-HIV effects.

## Risks and Benefits of VMMC

Although VMMC carries some risks, the benefits of VMMC exceed risks by 100- to 200-fold [10]. One of the largest benefits of VMMC is prevention of HIV acquisition, but other benefits include prevention of other sexually transmitted infections (STIs). This section briefly summarizes evidence regarding the risks and benefits of VMMCs.

### Reduction in Individual Risk of HIV

Currently, VMMC is the only one-time biomedical intervention to reduce lifelong HIV risk. Three randomized controlled trials conducted in Kenya, Uganda, and South Africa [1–3] estimated a 60% reduction in HIV infection risk for men in the 2 years following the recommended 6-week post-surgical abstinence period. Similar protection from HIV has been observed in post-trial follow-up and in community-based observational cohorts [11•]. Among men who have sex with men (MSM), the benefits of VMMC are less well-characterized and likely smaller in magnitude, estimated at approximately a 20% reduction in HIV acquisition on average, with heterogeneity depending on factors such as types of sexual encounters [12].

### Reduction in Community Risk of HIV

The HIV prevention benefits of VMMC extend beyond the recipient to the broader community. By avoiding infection, a circumcised man indirectly protects sexual partners, who in turn protect their sexual partners, and so forth. Mathematical modeling has suggested that HIV prevention benefits in ESA are approximately equally distributed between direct recipients and their communities over the first 5 years post-intervention. After 5 years, community benefits outpace individual benefits, especially when prevention recipients are male [13]. This is likely because, in generalized epidemics, women experience higher HIV prevalence and incidence than men, meaning that men are more often transmitters than recipients of new HIV infections [14].

### Reduction in Urological and Sexually Transmitted Infections

Reports of male circumcision reducing the risk of STIs date back to 1855 [15], well before the HIV pandemic. Modern studies have shown mixed results regarding the effects of circumcision on men’s acquisition of STIs [16–19] and transmission to sexual partners [20–22]. In meta-analyses, evidence strongly supports reduced risk of herpes simplex virus type 2 (HSV-2), human papillomavirus (HPV), and syphilis infections in circumcised men [23], although evidence is mixed for other STIs [24]. Female sexual partners of circumcised men have lower risk of HSV-2, human papillomavirus (HPV), *Trichomonas vaginalis*, and bacterial vaginosis [25], while sexual partners of MSM have lower risk of HSV-2 and HPV [26]. These effects are largest in populations with high overall prevalence of STIs [24].

Interestingly, reductions in HIV and STIs have also been observed in populations practicing dorsal longitudinal foreskin cutting, in which the foreskin is not removed, but a slit causes complete exposure of the underside of the foreskin and surface of the penile shaft [27]. Both the dorsal slit and full circumcision result in changes to the stratum corneum of the exposed skin [28] and to the composition and diversity of penile microbiota [29], although the precise mechanisms by which these changes reduce STIs are still under investigation.

## Risk of Adverse Events

Circumcision is a safe procedure when performed by trained personnel [30, 31] but like any surgical intervention, it carries a small risk of complications. Reported VMMC adverse events have been steadily declining in ESA [32, 33], although reporting is likely incomplete [34, 35]. There is an ongoing need to strengthen post-operative counseling and reporting [35–37] and maintain rigorous monitoring and evaluation [38].

Adverse event frequency differs by patient age [39]. Intraoperative adverse events are more common among patients older than 20 years [32]. Risk of infection is higher, but risk of bleeding is lower for ages 10–14 compared to 15 and older [34, 40–42]. A recent analysis by Lucas et al. [43•] found that boys age < 15 have a greater risk of rare but severe adverse events such as fistulas between the urethra and penile skin. These findings contributed to the 2020 decision by the U.S. President’s Emergency Plan for AIDS Relief (PEPFAR) to raise the minimum age for VMMC to 15 years.

### Risk Compensation

A common concern with HIV prevention is that recipients may increase sexual risk behavior, a phenomenon known as risk compensation. Gao et al. recently reviewed condom use and numbers of sexual partners among heterosexual men prior to 2015 and found that VMMC was not associated with increased risk-taking [44]. Several recent studies also failed to find evidence of sexual risk compensation after VMMC [45–49], while others found possible evidence of risk compensation [50–53] or risk trade-offs, such as increases in multiple sex partners combined with increases in condom usage [54]. Kwamba et al. performed a pooled analysis of VMMC randomized trials and cohort studies and found no risk compensation for at least 2 years post-VMMC [55•].

The effects of male circumcision on women’s risky behavior are less understood. Some studies suggest that women who are aware of male circumcision for HIV prevention may have a reduced perception of HIV risk, reduced condom usage, or greater number of sex partners [56, 57]. Further research is needed into the extent and possible mitigation of risk compensation among partners of circumcised men.

## VMMC Modeling Approaches

Estimating the long-term population-level impact of VMMC in ESA requires a glimpse into the future, which is typically obtained through mathematical modeling. Several models are being used to inform VMMC policy in ESA, each with different advantages and disadvantages.

### Scope of VMMC Models

Like any model, a VMMC model describes how a system — in this case, a human population — behaves over time. The model does not need to incorporate every nuance of the system, provided it captures the predominant features that govern important outputs [58]. VMMC models in ESA have mainly focused on how VMMC affects HIV-related morbidity and mortality, as well as its effect on healthcare spending — usually in the context of donor-funded HIV programs. Other effects of VMMC, including those discussed in the prior section, are rarely included in VMMC modeling (Table 1).

**Table 1:** Overview of health impacts of VMMC and their inclusion in VMMC modeling

Effect of VMMC	Impact	Magnitude	Usually incorporated into VMMC modeling?	Examples
HIV prevention (acquisition)	Positive	Large	Yes	[8, 59••, 60–66]
Healthcare budget	Negative for at least 13 years, then positive	Medium	Yes	[8, 59••, 60–62, 65••]
HIV prevention (transmission)	Neutral or positive	Unknown	Sometimes	[67, 68]
Risk compensation	Neutral or negative	Unknown	Sometimes	[68]
STI prevention	Positive	Medium	No	
Adverse events	Negative	Small	No	

The choice to focus on HIV-related outcomes assumes the predominance of HIV-related outcomes over other health effects of VMMC. This is plausible for ESA in the short term, as HIV is currently responsible for > 90% of the burden of all STIs in ESA [69]. In model projections where HIV burden declines by an order of magnitude or STI burden rises by an order of magnitude (or some combination of the two), STIs could become equally prominent factors for VMMC decision-making. Careful monitoring of VMMC safety, risk compensation, and accruing evidence on other effects will be needed to determine whether these additional factors should be included in VMMC modeling.

### Modeling Methods

For an HIV-focused VMMC model, the main goal is to translate VMMC-related changes in HIV susceptibility into projections of population-level HIV incidence, burden, and costs. The key features of these models are (1) HIV transmission, (2) progression of HIV disease, and (3) utilization of HIV services, especially cost-driving ones such as ART. These elements can be modeled in different ways, each with different advantages and disadvantages.

A representation of HIV transmission is needed to simulate the effect of VMMC. Individual-based models are able to simulate person-to-person transmission on a network (for example, the EMOD-HIV model [70]) and can also simulate group-to-group transmission based on collective attributes, such as average viral load (for example, the Synthesis HIV model [71]). Compartmental models, which represent categories of individuals, only simulate group-to-group transmission (for example, Goals [72], Optima HIV [9], and Thembisa [73]), with groups typically defined by age, sex, and key population or other proxy for risk level. Some VMMC models, such as the widely used the Decision Makers’ Program Planning Toolkit, Version 2 (DMPPT2) model [74], do not explicitly represent transmission at all, but instead “borrow” HIV incidence projections from a different model (Goals) and adjust these based on changes in male circumcision prevalence.

Representation of progression of HIV infection allows models to translate transmission reductions into changes in morbidity and mortality. Most models represent disease progression as a decline in CD4 + T-cell count, with lower CD4 counts leading to reduced quality of life due to AIDS-related complications and higher mortality rates. Age-structured models can then estimate the years of life lost due to HIV/AIDS. Models without age structure can also estimate this by approximating the average age at which an AIDS-related death is likely to occur.

Representation of HIV services is required to assess cost-effectiveness and budget impacts of VMMC, of which the most significant portion is treatment with ART. Minimally, such models must estimate the reduced need for ART due to HIV infections averted by VMMC, translating this into cost reductions over time. However, some models offer additional details such as ART drug resistance, non-adherence, treatment interruption, and delayed treatment initiation or re-initiation.

### Multi-model Analyses

In general, simpler VMMC models such as Goals and DMPPT2 have been applied more widely in ESA than complex models such as those incorporating age-structured transmission, patterns of engagement along the care continuum, and/or evolution of drug resistance. This is because simpler models tend to provide greater ease-of-use, interpretability of methods, and ability to be run without specialized computing equipment or expertise. However, it is not always clear which simplifications are acceptable in the context of evaluating VMMC programs. Multi-model analyses enable a head-to-head comparison of models evaluating the same policy options, with the goal of determining whether the estimates and policy recommendations are similar or different across model structures and assumptions.

Ideally, a multi-model analysis includes models with diverse structures, assumptions, and ways of fitting epidemic data. It is critical that model comparisons not create “groupthink” by discarding potentially valid model structures and assumptions for the sake of reaching agreement. However, the models should align on the specific policies and scenarios under evaluation. Korenromp et al. recently undertook a model comparison of three models of VMMC in South Africa with diverse structures: Goals (simpler compartmental), Thembisa (more complex compartmental), and EMOD (complex agent-based/network). The analysis aligned on the number of VMMCs performed by age and geography, the cost per circumcision and per year of ART, and the time horizon and annual discount rate for the analysis, and found similar estimates for VMMC impact on HIV transmission, burden, and healthcare budgets across the diverse model structures [59••].

## Cost and Cost-effectiveness of VMMC

VMMC has generally been considered one of the most cost-effective HIV prevention options available in ESA. However, questions have been raised as to whether shifts in the HIV epidemic might change VMMC cost-effectiveness or have implications for optimal ways in which VMMC resources should be distributed. Here we review evidence and current limitations in assessing the long-term cost-effectiveness of VMMC in ESA priority countries.

### VMMC Costs

Numerous studies have attempted to quantify the cost of VMMC in ESA priority countries. Most studies have focused on service delivery costs, such as personnel, capital costs of buildings and equipment, and recurrent costs of rent, maintenance, and consumable goods [75]. Pineda-Antunez et al. compared ESA country averages for service delivery costs per VMMC delivered and found significant variation by country income level, with low-income country facilities spending a mean of US$42 per VMMC, while upper-middle income country facilities spent a mean of US$114 per VMMC [76•]. In most countries, the largest cost component was personnel, and higher salary levels tend to drive differences in costs.

The validity of a country-level unit cost for VMMC has been challenged because, within a country, unit costs vary greatly across facilities. Bautista-Arredondo et al. [77] found that much of the variation in VMMC cost can be predicted by a facility’s attributes, with lower-volume facilities, hospitals, and privately owned facilities incurring higher unit costs, while urban versus rural location did not independently predict unit costs. This study estimates that a 10% increase in facility volume could reduce cost per VMMC by 12–22%.

An important limitation of these studies is that they rarely include indirect costs incurred above the facility level, such as informational campaigns, program operations, and monitoring and evaluation [78]. One study estimated that in South Africa, over US$14.2 million was spent in 2014 on VMMC outreach efforts such as hiring community mobilizers and carrying out media campaigns [78]. Mangenah et al. found that in Zimbabwe, adding human-centered design (HCD) approaches to standard VMMC demand creation lowered the cost per VMMC, but further addition of HIV self-testing services increased the cost per VMMC [79••].

In addition to health system costs, VMMC incurs some out-of-pocket costs for patients. In South Africa, transportation was the largest out-of-pocket cost for VMMC clients, costing US$9.20 per client on average, or roughly 7–8% of the VMMC unit cost [80]. These costs are not included in economic analyses from the health system perspective, but are useful for analyses from the broader societal perspective.

### VMMC Cost-effectiveness

VMMC has been estimated in many settings to reimburse the healthcare system through treatment costs avoided over the recipient’s lifetime, even before taking into account health benefits. Korenromp et al. used three HIV models to assess the budgetary impact of the South African VMMC program implemented in 2009–2017. In all models, VMMC costs would be reimbursed by the savings from averted treatment around 2034–2039 [59••]. These findings are consistent with earlier analyses in South Africa, which estimated that the costs of a given circumcision could be amortized over as little as 12 years (for a 25-year-old) or as much as 23 years (for a 10-year-old) [60].

**Fig. 1:**
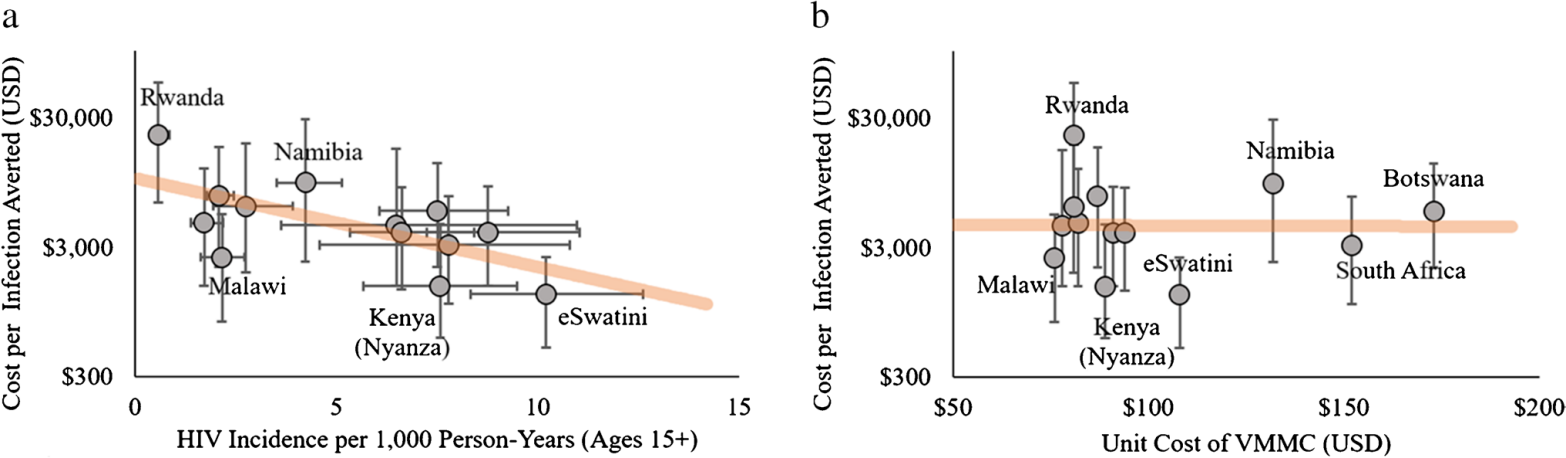
Association between cost per HIV infection averted by VMMC and **a** HIV incidence or **b**unit cost of VMMC. Unit costs and costs per infection averted were obtained from a study by Kripke et al. [65••] which used direct costs and modeled impact of VMMCs performed over 2010–2025 for each country except for Kenya where only the Nyanza region was included. Incidence was obtained at the national level from UNAIDS [81] and for Nyanza from three published estimates [13, 82, 83]

VMMC cost-effectiveness estimates have tended to be consistent across models of the same setting, but widely different across ESA settings. Korenromp et al. found a cost of $1070-1220 per HIV infection averted for the South African VMMC program, a remarkable agreement across three South Africa HIV models with widely different structures [59••]. In an analysis of 14 ESA priority countries, Kripke et al. found that costs per infection averted ranged from US$1300 to $22,000 [65••]. The variability was driven more by the wide differences in HIV incidence across ESA settings [13, 81–83] (Fig. 1a) than by differences in unit costs of VMMC (Fig. 1b). The analysis suggests that providing VMMC in high-incidence countries is more important than reducing costs. A limitation of this analysis is that it approximated above-facility costs as a 15% overhead on service delivery costs in all countries, although it is likely that demand generation costs vary by country and over time as more demand is satisfied.

### Budgeting for VMMC

Although VMMC is eventually cost-saving in much of ESA, health agencies cannot always afford to “lend” to VMMC programs in the short term when there are competing health resource needs. Internationally funded HIV programs typically consider an intervention to be cost-effective if it costs less than ~ US$500 per DALY averted or QALY gained, after applying an annual discount rate of at least 3% [84, 85]. Many ESA VMMC programs meet these cost-effectiveness benchmarks. For example, Kripke et al. found that Malawi’s VMMC program costs US$120 per DALY averted in urban areas and US$355 per DALY averted in rural areas [86•], both of which would be considered cost-effective within internationally funded HIV programs.

However, in the event that international donors do not cover the costs of VMMC programs, domestic funding for VMMC is much more constrained in lower-income countries. In Malawi, it is estimated that only services costing less than US$130–164 per DALY averted would be a cost-effective use of limited domestic health spending [87, 88]. In this case, only urban VMMCs would be considered cost-effective in Malawi. In contrast, higher-income economies in ESA face less stringent budget constraints. In South Africa, it is estimated that domestic health spending is cost-effective below US$3525 per DALY averted [89], making VMMC highly cost-effective [5, 90].

Another approach to estimating cost-effectiveness is an analysis that optimizes the allocation of a fixed budget across HIV services. In budget optimization analyses, VMMC has consistently been recommended for funding in South Africa [4, 5], Kenya [6], Zimbabwe [7, 8], and elsewhere in ESA [9], usually rising to the top of budget prioritization due to its cost-effectiveness. The recommended budget allocation for VMMC is usually smaller than for ART, however, due to the higher lifetime cost of ART compared to VMMC, and the immediacy of ART health benefits compared to more delayed benefits from VMMC.

## Subnational Allocation of VMMC

We have seen how the impact and cost-effectiveness of VMMC can vary widely across ESA countries. It is therefore logical that VMMC cost-effectiveness varies subnationally. These differences have been evident in recent subnational estimation of VMMC impact and cost-effectiveness.

### Geographic Prioritization of VMMC

The relationship between incidence and VMMC impact on a national level (Fig. 1) suggests that there would be a strong relationship between incidence and VMMC impact across subnational geographic areas. This relationship has indeed been evident in subnational VMMC modeling, but despite subnational variation, VMMC was found to be cost-saving in all regions of South Africa [91] and Uganda [92], and all 11 VMMC priority regions of Tanzania [93]. In Malawi, VMMC was found to be cost-saving in the South West and South East health zones and cost-effective but not cost-saving in the Northern and Central West zones [86•]. VMMC was not found to be cost-effective in the Central East zone where Lilongwe is located, but when the geographies were split up by urban versus rural rather than by zone, VMMC in urban areas was found to be cost-saving, providing a rationale for continuing the already-established VMMC program in Lilongwe.

Subnational geographic analyses were confounded by wide uncertainty ranges in the HIV incidence projections, leading to wide and often overlapping uncertainty ranges for VMMC cost-effectiveness by geography. Improvements in methods for estimating HIV incidence with greater geographic granularity, including tests for recent HIV infection [94] and geospatial “big data” methods [95], are poised to enable more sophisticated geographic prioritization of VMMC.

### Age-Based Prioritization of VMMC

In the early years of VMMC scale-up, a vast majority of VMMC clients were adolescent boys under age 15, while the WHO/UNAIDS VMMC coverage targets had been set for ages 15–49. This raised questions about how best to prioritize VMMC by age. In response, age-based prioritization was examined in a multi-model analysis spanning nine ESA countries [96].

The ASSA2008 model of South Africa showed the greatest financial savings (US$617 per circumcision) when circumcising 20-year-olds, assuming a discount rate of 5% over a 45-year timeframe [90]. In a multi-model comparison, two models, ASM and DMPPT2, used harmonized assumptions about VMMC costs, discount rates of 3% per year, and a time horizon of 15 years. DMPPT2, applied in eight countries, found the largest number of HIV infection averted per VMMC over 15 years in the 20–24-, 25–29-, and 30–34-year age groups [74], while ASM, applied in Zambia and Zimbabwe, found the highest number of infections averted in the 15–19-, 20–24-, and 25–29-year age groups [67]. An important driver of these differences was the age distribution of HIV incidence, which was highest in men ages 20–29 in ASM and ages 25–34 in DMPPT2.

An important limitation of these cost-effectiveness analyses is the lack of data on how VMMC cost varies by client age. Demand creation tends to become more challenging at older ages. Kripke et al. [61] attempted to address this with fictitious scenarios about the relative costs of circumcising adolescent versus adult clients in Zimbabwe, finding that a 50% higher unit cost for adults compared to adolescents would make VMMC more cost-effective in adolescents than adults. Torres-Rueda et al. [97•] conducted a randomized, controlled trial comparing VMMC with and without strategies to generate demand among 20–29-year-olds. VMMC with demand creation intervention was found to be more cost-effective overall, but the intervention did not preferentially increase uptake among adults compared to adolescents, highlighting the challenge of generating demand in older age groups.

### The Future of VMMC in ESA: Opportunities and Challenges

ESA countries are now striving toward the Sustainable Development Goal of HIV epidemic control by 2030 [98], an effort in which VMMC could play a substantial role. In Uganda [99], Kenya [100], and elsewhere, attainment of high VMMC coverage has been associated with declines in HIV incidence, and model-based predictions suggests that many more infections will be averted over the coming decades by VMMCs already performed [59••]. Modeling further predicts that achieving 90% VMMC coverage would avert as many HIV infections as attainment of the UNAIDS 90–90-90 HIV treatment goals [62]. However, most of ESA is far behind VMMC targets [101], despite many areas approaching or surpassing the 90–90-90 treatment targets [102]. The totality of this evidence suggests that VMMC remains an underutilized opportunity to accelerate HIV epidemic control.

It comes as little surprise that VMMC, even when highly cost-effective as an HIV intervention, is more difficult to advocate for than more direct interventions such as ART. Millions of PLHIV are almost certainly alive today thanks to ART. With VMMC, it is impossible to point to a specific HIV-negative person and know that this particular person remained negative because of VMMC. Additionally, most of the benefits of VMMC accrue over many years. Even though cost-effectiveness calculations take into account discounting over time, the long wait times further obstruct the visibility of VMMC’s benefits. Decision-makers must rely on models, rather than direct observation, in order to motivate prioritizing VMMC — which, in turn, requires credibility, visibility, and trust in VMMC modeling.

As we have discussed, there are many types of VMMC models, and multi-model analyses can be used to test the robustness of VMMC estimates to differences in model structures and assumptions. However, even multi-model analyses can fail to adequately guide VMMC decision-making when models provide conflicting results, or when all participating models use similarly flawed assumptions. For example, models provided conflicting results regarding optimal age groups for VMMC, even after harmonizing assumptions about time horizons and discount rates. Improving the accuracy and precision of age-specific HIV incidence estimation could help to resolve these differences. VMMC modeling is also susceptible to use of common flawed assumptions, especially when models rely on common and imperfect data sources for key inputs such as population sizes and VMMC coverage levels [103]. Demand generation costs, especially how they change across population groups and levels of VMMC coverage, are a particularly important data gap.

Finally, few VMMC models consider impacts of VMMC other than direct reductions in HIV acquisition. However, as HIV incidence declines, other impacts will become non-negligible, and models will need to adapt to represent VMMC impacts more comprehensively.

## Conclusions

Although VMMC remains one of the most efficient HIV interventions available in ESA, its benefits are not as visible as other HIV interventions, such as ART. Mathematical models are needed to estimate the impact and cost-effectiveness of VMMC, often as a multi-model analysis to check the robustness of estimates to different model structures and assumptions. Recent multi-model analyses have confirmed the cost-effectiveness of VMMC in most ESA settings, especially those with high HIV incidence. However, some disagreement remains regarding the optimal age groups to receive VMMC, and model projections could be improved by filling key data gaps, such as demand generation costs across populations and time. Despite these limitations, modeling has provided crucial input into VMMC strategic directions [104] and continues to reinforce the importance of VMMC in ESA.
